# Reduced order modeling and model order reduction for continuum manipulators: an overview

**DOI:** 10.3389/frobt.2023.1094114

**Published:** 2023-09-15

**Authors:** S.M.H. Sadati, S. Elnaz Naghibi, Lyndon da Cruz, Christos Bergeles

**Affiliations:** ^1^ Robotics and Vision in Medicine (RViM) Lab, School of Biomedical Engineering and Imaging Sciences, King’s College London, London, United kingdom; ^2^ Department of Aeronautics, Faculty of Engineering, Imperial College London, London, England, United kingdom; ^3^ Wellcome/EPSRC Centre for Interventional and Surgical Sciences, University College London, London, England, United kingdom; ^4^ Moorfields Eye Hospital, London, United kingdom

**Keywords:** soft robot, continuum robot, reduced order model (ROM), dynamics, model order reduction (MOR)

## Abstract

Soft robot’s natural dynamics calls for the development of tailored modeling techniques for control. However, the high-dimensional configuration space of the geometrically exact modeling approaches for soft robots, i.e., Cosserat rod and Finite Element Methods (FEM), has been identified as a key obstacle in controller design. To address this challenge, Reduced Order Modeling (ROM), i.e., the approximation of the full-order models, and Model Order Reduction (MOR), i.e., reducing the state space dimension of a high fidelity FEM-based model, are enjoying extensive research. Although both techniques serve a similar purpose and their terms have been used interchangeably in the literature, they are different in their assumptions and implementation. This review paper provides the first in-depth survey of ROM and MOR techniques in the continuum and soft robotics landscape to aid Soft Robotics researchers in selecting computationally efficient models for their specific tasks.

## 1 Introduction

Robots with continuum structures can mimic highly dexterous and deformable biological bodies, follow curved paths through body lumens (e.g., vessel interiors) in minimally invasive medical interventions, enable safe interactions with the environment (Rus and Tolley, 2015). Considering compliant structures, however, brings forth issues such as uncertain deformations, limited control feedback, reduced control bandwidth and slow response, stability problems, underdamped modes causing persistent vibrations, and lack of precision under external loads (Cianchetti et al., 2013; Blanc et al., 2017).

Soft Roboticists have approached such challenges by proposing new theoretical frameworks and tailored software packages (Sadati et al., 2020a). On one hand, geometrically exact Cosserat rod Variable Curvature kinematics and Finite Element methods (FEM) have proven to be of high fidelity (Trivedi et al., 2008; Coevoet et al., 2017; Grazioso et al., 2019), but bring forth computational obstacles when used in real-time applications, such as control, are considered. On the other hand, Reduced Order Modeling (ROM) and Model Order Reduction (MOR) techniques, where low-dimensional continuous or discreterepresentations are used to approximate the full-order system (i.e., variational) kinematics representation or FEM solution for a continuum structure, can both address modeling challenges and improve computational performance (Book, 1990; Della Santina et al., 2110; Goury, Duriez). Such methods have pioneered methods for flexible-link structures (Book, 1990) and highly articulated (Chirikjian, 1993) robots and, more recently, have been permeating the soft robotics community (Goury, Duriez; Sadati et al., 2018; Singh et al., 2018).

Although Reduced Order Modeling and Model Order Reduction terms are utilized interchangeably in the literature, this paper makes a distinction and highlights their different assumptions, implementation steps, and capabilities, in order to help with deciding the most appropriate method for a given application. To this end, as instances of Reduced Order Modeling, we review discretization-based, modal, and strain techniques, where the continuum kinematics is constructed based on simplifying assumptions. Alternatively, schemes where the high dimensionality of a continuum robot is reduced, e.g., neglecting the less dominant deformation modes in a FEM solution, are considered as instances of Model Order Reduction. In this paper, we don’t consider reduced order integration schemes, e.g., (Boyer et al., 2020; Godage et al., 2016; Caasenbrood et al., 1089), which may simplify the derivation of the dynamics without necessarily reducing the modeling state space.

Detailed review papers have recently discussed modeling of continuum robots and slender rods, and readers, novice or expert, can find details on modeling assumptions therein (Della Santina et al., 2110; Rao et al., 2020; Gilbert, 2020; Sadati et al., 2017a; Armanini et al., 2023). Our paper instead provides a classification based on classic continuum mechanics that highlights how to combine assumptions for a soft robot kinematics, governing equations, material mechanics, and solution strategies. More specifically, we reflect on the Reduced Order modeling techniques, as an emerging trend in the Soft Robotics community, and highlight the need for further studies to explore their full potential. Our goal is to provide a “recipe” that supports researchers, with moderate knowledge of the field and an interest in Reduced Order Modeling techniques, in identifying the most suitable modeling assumptions and solution strategy for their application.

The rest of this article is organized as follows. A brief review of the necessary elements of a soft robot model is discussed in Section 2. A detailed review of Reduced Order Modeling and Model Order Reduction methods and complementary states space coordination and solution strategies is provided in Section 3. The main features, advantages, and shortcomings of their different implementations are listed and contrasted in Tables 2–5. The manuscript concludes in Section 4. Table 1 lists the acronyms in literature, although we mostly use their expanded from in the rest of this manuscript.

**TABLE 1:** Acronyms used in the literature for the simplified kinematic representation of a continuum robot.

Acronym	Explanation	Acronym	Explanation
ANCF	Absolute Nodal Coordinate Formulation	PRB	Pseudo Rigid Body assumptions
CC	Constant Curvature	PS	Polynomial Shape parametrization
FEM	Finite Element Method	PVC	Piecewise Variable Curvature
MOR	Model Order Reduction	PVW	Principle of Virtual Work
PC	Polynomial Curvature parametrization	ROM	Reduced Order Model
PCC	Piecewise Constant Curvature	VC	Variable Curvature

## 2 Soft robot modeling: assumptions and solution strategies

This section categorizes the key elements of a continuum manipulator model from continuum mechanics theory point of view. This is to better understand how Reduced Order Modeling techniques fit in the soft manipulator modeling framework. The two key elements in theoretical modeling of a soft robot (except for deep learning approaches) are:

I. the *modeling assumptions* for:
  A. system *kinematics*,
  B. system *mechanics* (*governing equations* or *conservational law*),
  C. *material mechanics* (*constitutional law*); and
II. the *solution strategy* (direct or indirect) for the resultant system of differential equations.

In the rest of this paper, we utilized the term “state space” to refer to the time-variant states of a continuum manipulator model. This is to distinguish between the time-variant states of a Reduced Order model (e.g., modal contribution or fitting function coefficients) with the robot configuration (e.g., strains, position, and orientation) and joint (e.g., joint angles and displacements in a Pseudo Rigid Body model) spaces.

### 2.1 Continuum robot kinematics

The kinematics of a continuum robot can be represented based on:

1. *Full-order* representations, i.e.,:
  a. *Variable Curvature* (VC), also called Continuum, Differential, Cosserat Rod, Geometrically Exact Kinematics (Trivedi et al., 2008; Rucker and Webster, 2011; Till et al., 2019),
2. *Shape (Basis) function-based,* also called Geometrical Kinematics (Armanini et al., 2023), solutions that approximate the bending angle (Gravagne and Walker, 2000; Gravagne and Walker, 2002; Gravagne et al., 2003; Gravagne and Walker, 2012), strain field (Orekhov and Simaan, 2008; Della Santina et al., 2020a; Della Santina and Rus, 2020) or shape (Godage et al., 2011a; Godage et al., 2011b; Godage et al., 2011b; Godage et al., 2015a; Sadati et al., 2018; Singh et al., 2018; Sadati et al., 2020a; Rao et al., 2022) of the entire robot backbone via modal or fitting approaches,
3. *Discretized* representations, i.e.,:
  a. *Pseudo Rigid Body* (PRB) approach that approximates the robot kinematics as a highly-articulated serial rigid-link mechanism with states defined in relative coordinates, i.e., strains as rotation and *translation* of rigid-robot joints (Howell and Midha, 1995; Howell et al., 1996; Yu et al., 2005; Sadati et al., 2017a; Shiva et al., 2019; Sadati et al., 2020a; Rao et al., 2020; Schultz et al., 2022),
  b. *Piecewise Constant Curvature* (PCC) that discretizes the backbone as a series of Constant Curvature infinitesimal segments with states defined in relative coordinates (Sadati et al., 2020a; Caasenbrood et al., 1089; Rao et al., 2020; Shiva et al., 2019; Renda et al., 2018; Renda, Giorgio-Serchi, Boyer, Laschi, Dias, Seneviratne),
  c. *Piecewise Variable Curvature* (PVC), with basis function-based (e.g., a polynomial) estimation of curvature value along each segment (Boyer et al., 2020), or
  d. *Absolute Nodal Coordinate Formulation* (ANCF), based on position and orientation of finite number of nodes along the backbone as the system states defined in absolute coordinates (Shabana, 2018; Sadati et al., 2020a; Huang et al., 2021; Shabana and Eldeeb, 2021).

*Constant Curvature* (CC) kinematics can considered as a special case of basis function-based fitting for a continuum segment (Webster and Jones, 2010; Mustaza et al., 2019; Della Santina et al., 2020b). In the case of Piecewise Constant Curvature kinematics, the transformations between the segments can be derived based on rotation matrices (Sadati et al., 2017a; Shiva et al., 2019), quaternions (Sadati et al., 2020a), or screw theory (Renda et al., 2018; Renda, Giorgio-Serchi, Boyer, Laschi, Dias, Seneviratne). The underlying transformation can be based on Constant Curvature assumptions (Sadati et al., 2017a; Sadati et al., 2017b; Della Santina et al., 2020b; Rao et al., 2020), discretization of Cosserat Rod (i.e., Variable Curvature) kinematics (Renda et al., 2018; Shiva et al., 2019; Sadati et al., 2020a), or deformation map of an Euler-Bernoulli beam (Sadati et al., 2020a). In the case of Absolute Nodal Coordinate Formulation, such transformations can be based on a basis function such as a polynomial (Huang et al., 2021) or deformation map of an Euler-Bernoulli beam (Sadati et al., 2020a). Furthermore, fitting solutions are proposed for approximating the kinematics of a soft robot cross section geometry deformation, showing 1%–3% increase in the accuracy of a pneumatic soft appendage known as STIFF-FLOP (Sadati et al., 2017b).

**FIGURE 1:**
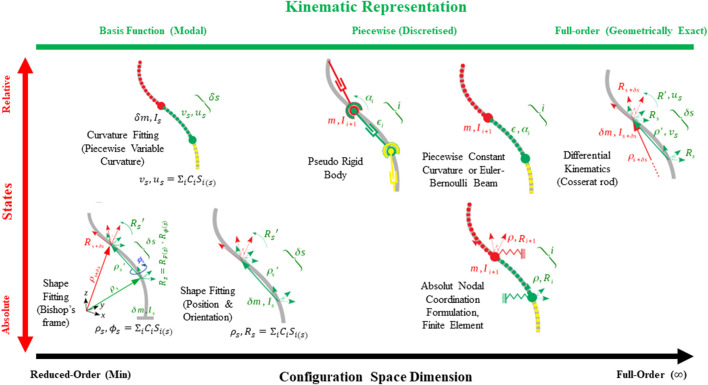
Different kinematics representations for a continuum manipulator in Soft Robotics literature. Here R and p denote the local frame rotation matrix and position vector, m and I are the mass and inertia, v and u are the local translational (shear and elongation) and rotational (bending and torsion) strains, ϵ and α are the translation and Euler angular rotation of an element in the local frame, s is the unit length along the backbone, C and S are the coefficient and basis function for the basis function-based kinematics, i is a free index, δ and ′ are operators denoting infinitesimal change and partial differentiation along and w.r.t. s .

Variable Curvature (also referred to as *Continuum* representation (Armanini et al., 2023) provides a geometrically exact kinematic representation at the cost of a system with infinite number of states not suitable for controller design. Basis function-based methods present a low-dimensional continuous formulation suited for controller design with high modeling accuracy, however, are prone to simulation errors. The Pseudo Rigid Body method enables the usage of rigid-body robotic modeling and control techniques for flexible and continuum robots but is usually applicable for a small system (with small number, less than five, number of discretized segments) and usually suffers from singularity issues. This is due to the exponential growth of the derivations for the distal segments in a series rigid link mechanism. The Piecewise Constant Curvature techniques improve the modeling accuracy of a Pseudo Rigid Body representation by considering the structure deformation as a circular arc with limited applicability to large systems (with large number of discretized segments) and with external or body loadings. The Piecewise Variable Curvature technique provides a better estimate of the beam deformation with a polynomial curvature representation but is still not applicable to large systems due to the complex derivations for distal segments, similar to the case of the Pseudo Rigid Body method. Absolute Nodal Coordinate Formulation is the most accurate method for modeling a large system, via optimal sparse matrix formulation of the system dynamic equations, and for incorporating different material mechanics models and actuation forces (Shabana, 2018), at the cost of a large state space dimension and complex models for internal forces and actuation inputs. Figure 1 presents the different kinematic representations for a continuum manipulator in the Soft Robotics literature.

### 2.2 System mechanics (governing equations) derivation

The *system mechanics* can be derived for:

1. *Euler-Bernoulli Beam Theory* (static), i.e., based on Euler-Bernoulli beam formulation (Wegiriya et al., 2019; Yu et al., 2021),
2. *Cosserat rod* method (static and dynamic) (Trivedi et al., 2008; Rucker and Webster, 2011; Burgner-Kahrs et al., 2015; Sadati et al., 2017a; Sadati et al., 2017b; Gazzola et al., 2018; Till et al., 2019; Rao et al., 2020),
3. *Principle of Virtual Work* (PVW, static and dynamic) (Sadati et al., 2017a; Sadati et al., 2017b),
4. *Lagrangian Formulation* (dynamic) based on shape function-based kinematics (Godage et al., 2016; Mustaza et al., 2019; Della Santina et al., 2020a; Della Santina and Rus, 2020), or discretized kinematics (Godage et al., 2015b; Sadati et al., 2020a; Della Santina et al., 2020b; Habibi et al., 2020; Schultz et al., 2022),
5. *TMT Dynamics* (dynamic) (Sadati et al., 2018; Sadati et al., 2019a; Sadati et al., 2020a; Sadati et al., 2022), which is the vector format for the Principle of Virtual Work that derives the system dynamics using a vector formalism with fewer derivation steps compared to the Lagrangian approach, and
6. *Recursive Computation Scheme* (dynamic) (Godage et al., 2016) (also called *Reduced Order Integration* scheme (Caasenbrood et al., 1089) or *Inverse Dynamic Model* (Boyer et al., 2020), for forming the equation of motion in a vector formalism by recursively evaluating the nonlinear equations of motion based on Lagrangian or Cosserat rod dynamics.

Euler-Bernoulli Beam theory provides the simplest mechanics formulation for a continuum robot but is only applicable to small deformation cases. Cosserat rod mechanics provides an exact solution in the form of a boundary value problem, known to be computationally expensive to solve via numerical methods. The Principle of Virtual Work is a powerful method of deriving governing equations to incorporate a robot structural complexity and inhomogeneities but challenging to derive for dynamic cases. Lagrangian dynamics (i.e., derivation of the equations of motion using Lagrangian methods) is the commonly utilized method for dynamic cases but results in complex nonlinear relations that are hard to rearrange in a vector formalism, i.e., linear w.r.t. the acceleration terms, when using a standard symbolic mathematical software, e.g., Matlab. Identifying repetitive structures in the Lagrangian formulation can simplify this problem (35).

TMT and Recursive Computation Scheme are proposed to derive such a form. TMT method, which is named after the formulation of the generalized mass matrix in a dynamic system (Sadati et al., 2020a), is an easy-to-interpret analytical derivation for the system dynamic equation of motion via direct evaluation of the terms in a vector format. However, it can be analytically challenging to derive the vector format of the virtual work associated with some of the terms in a continuum system dynamics. It is worth noting that the Principle of Virtual Work, Lagrangian, and TMT Dynamic formulations result in the same set of final derivations, but with different derivation steps. The Recursive Computation Scheme simplifies the vector-format formulation of a complex dynamic model based on the numerical evaluation of the equation of motion terms by selectively setting the inertial, damping, gravitational, and elastic terms to zero. As a result, it is more computationally expensive due to multiple recursive numerical evaluations of the governing equations.

### 2.3 Material mechanics (constitutional law)

The *material mechanics* for a continuum robot is usually derived based on:

1. *Hooke’s law* (Trivedi et al., 2008; Rucker and Webster, 2011; Godage et al., 2016; Sadati et al., 2017a; Sadati et al., 2017b; Till et al., 2019) (i.e., linear elasticity, infinitesimal strain theory) for elastic materials (e.g., Nitinol),
2. *Finite strain theory*, for hyper-elastic materials (e.g., rubber and silicon), based on methods such as:
  a. *Neo-Hookean* (Trivedi et al., 2008; Sadati et al., 2017a; Sadati et al., 2017b; Shiva et al., 2019),
  b. *Mooney-Rivlin* (Gopesh and Friend, 2020),
  c. *Gent* (Shiva et al., 2019), or
3. *Hyper-Viscoelastic* models, when viscosity matters (e.g., real tissue), based on methods such as:
  a. *Non-Newtonian fluid* via viscosity power law (Sadati et al., 2018; Mustaza et al., 2019; Sadati et al., 2020a) and
  b. *Kelvin-Voigt* method (Mustaza et al., 2019).

Hooke’s law is the commonly used material model with acceptable accuracy for slender systems with small strains. Finite strain theory is used for cases experiencing large strains but cannot capture the material hysteresis and creep effects. The neo-Hookean method is the standard technique for hyper-elastic materials but with limited accuracy in practical cases. Mooney-Rivlin method offers a more accurate model with a standard identification procedure but is still limited to strains less than 100%. The Gent method is the standard technique for larger strain cases and is commonly used for modeling rubber structures. Modeling the material hysteresis is possible by considering a hyper-viscoelastic model. The viscosity power law is a simple method but is more suitable for fluid mediums. The Kelvin-Voigt method can provide a good estimate of the material behavior with a standard process to identify the material properties. The nonlinear formulation of hyper-elastic and hyper-viscoelastic models makes their implementation challenging in a continuum robot model. The Principle of Virtual Work is the most powerful method of deriving the system constitutional law to incorporate different material mechanics representations in a continuum robot model.

### 2.4 Model formation and solution

A variety of combinations of the above choices for the system kinematics, mechanics, and material mechanics are possible to model a continuum robot. As a result, a system of Ordinary (ODE, mostly for dynamic models with discretized or reduced order kinematics) or Partial Differential Equations (PDE, mostly for quasi-static models, most forms of FEM, and Cosserat rod dynamics) is formed to be solved numerically considering the system’s initial and/or boundary conditions. An ODE formulation is possible for a model with variable curvature kinematics and Cosserat rod mechanics with general loading if load Boundary Conditions (e.g., moment, shear, and tension) are known (Xu et al., 2013; Sadati et al., 2020b) or for quasi-static models based on discretized kinematics in the absence of distributed, body, or external loads (Shiva et al., 2019).

The common solution strategies, as for any dynamic system, are:

1. *Direct (forward) integration* schemes (e.g., Runge-Kutta) for ODEs, via:
  a. Temporal (time-domain) discretization & integration of a dynamic model (most common) (Godage et al., 2015b; Godage et al., 2016; Sadati et al., 2020a), or
  b. Spatial (spatial-domain, e.g., along the backbone curve) discretization and integration of a dynamic model (Till, 2019; Till et al., 2019; Till et al., 2019; Till et al., 2020) or a static model (special cases as explained above) (Xu et al., 2013; Shiva et al., 2019; Sadati et al., 2020b);
2. *Indirect* schemes, commonly used for BVPs, via:
  a. *Shooting* (optimization-based) methods for static or Cosserat rod dynamic models which relies on guessing the solution vector and Jacobian-based numerical optimization techniques (Godage et al., 2011a; Rucker and Webster, 2011; Sadati et al., 2017a; Sadati et al., 2017b; Sadati et al., 2018; Aloi and Rucker, 2019; Till et al., 2019; Sadati et al., 2020a),
  b. Ritz-Galerkin (Tunay, 2013; Sadati et al., 2018) method for basis function-based solutions where the system of equations is weighted (i.e., multiplied) by the basis functions vector to achieve higher estimation accuracy.

Direct methods are numerically more efficient because they do not rely on an initial guess, but they are limited to system models in the form of ODEs. Temporal domain integration is the standard technique used in the community but may suffer from time-stepping issues for systems with highly oscillatory states. Spatial domain integration techniques can better handle a system with highly oscillatory states but are not as developed as the temporal domain integration schemes. Indirect schemes are suitable for geometrically exact models that are usually in the form of a BVP. Shooting methods are the standard methods to solve such systems at the cost of computational performance, resulting in slow simulations. The Ritz-Galerkin method can turn a BVP into an ODE to lower the simulation computational cost at the cost of a more complex derivation, challenges with selecting suitable basis functions, and convergence guarantee studies.

### 2.5 Data-driven and learning-based models

Although data-driven techniques do not necessarily fit in our presented categories above, a short survey of their advantages and limitations can help better justify the usage of Reduced Order Modeling techniques for soft manipulators. Analytical models for continuum robots present a robust solution in the presence of unknown conditions. However, it is not easy to capture commonly observed phenomena in soft robot real behavior such as fabrication inconsistencies, visco-hyper-elastic material properties, material creep, and hysteresis. Learnt solutions (when trained) are on the other hand real-time, accurate (within the training dataset domain), capable of handling complex structural geometries and nonlinearities, and easy to implement by not requiring significant knowledge of continuum robot theory (Thuruthel et al., 2017; Thuruthel et al., 2018a; Jolaei et al., 2020; Truby et al., 2020; Kim et al., 2021; Wang et al., 2021; Wang et al., 2022). However, the validity of a deep learning-based solution without a conservational law is limited to the richness and generality of the dataset used for learning, hence lacking robustness in presence of new conditions, contacts, and external loads which are not tested before (i.e., available in the dataset). Lack of generality and robustness guarantee are the main drawbacks of data-driven methods.

The learning-based solution drawbacks limit the usability of such methods to the case of improving a soft robot modeling accuracy for repetitive tasks but challenging to apply in highly varying and dynamic environments such as medical surgery. Koopman operator techniques (Bruder et al., 2019; Bruder et al., 2021a; Bruder et al., 2021b), learning the residual (unmodelled) system dynamics, and a combination of learning-based forward models with analytical controller designs can address some of these issues (Della Santina et al., 2110). Alternatively, training such models based on high-fidelity analytical or FEM-based models may solve the robustness and lack of generality issues of data-driven approaches by providing them with rich enough datasets that are hard to gather with a real robot (Iyengar et al., 2020; Iyengar and Stoyanov, 2021). A more detailed review of the topic is presented in (Della Santina et al., 2110; Thuruthel et al., 2018b). The rest of this paper provides a review of Reduced Order Modeling and Model Order Reduction techniques for modeling soft manipulators.

## 3 Reduced order modeling and model order reduction

Any effort to approximate the differential evolution of states, i.e., differential kinematics of a Cosserat rod, in terms of a representation with finite number of states, e.g., Pseudo Rigid Body kinematics, is often called Model Order Reduction approach in classic continuum mechanics. Although commonly employed for FEM (Tunay, 2013), Computational Fluid Dynamics (Naghibi et al., 2019), and in control theory (Moore, 1981; Antoulas, 2005; Van Dooren et al., 2008), the application of lightweight slender rigid manipulators for space explorations, undergoing infinitesimal deformations, has motivated the usage of Model Order Reduction techniques in robotics research since 1980s (Book, 1990). More recently, Model Order Reduction (also referred to as Reduction or, interchangeably, as Reduced Order Modeling) techniques are utilized for Soft Robotics research to capture the complex dynamics of active highly deformable structures. In this section, we follow the naming conventions used by (Thieffry et al., 2018a; Sadati et al., 2018) in which Reduced Order Modeling refers to the methods that construct a reduced order kinematics for a soft robot, while Model Order Reduction is used when compression techniques are utilized the reduce the state space dimension of a FEM model.

The main features, advantages, and shortcomings of the different implementations of Reduced Order Modeling and Model Order Reduction are presented and compared with each other in Table 2. A brief review of these methods in Soft Robotics research is presented in this section. Relevant coordinate formulations and solution strategy are discussed at the end of this section and summarized in Tables 3, 4, respectively.

**TABLE 2:** Reduced Order Models and Model Order Reduction for continuum and soft robots in literature. SF is Shape (Basis) Function.

Categories	Model order reduction (MOR) (compression)		Reduced order modeling (ROM)				
Solution	FEM		Modal solution, then Separation of variable				
EOM	Discretized		Continuous ODE				
SF Name	Principle Components (Modes)		Mode Shapes				
SF Selection	Principal Component Analysis		Eigenvalue analysis	Assumed modes (shape functions), randomly or based on experimental result fitting			
Reduction Method	Principal Orthogonal Decomposition		Linear Weighted Summation	Spectral (e.g., Fourier)	Power series (e.g., Taylor)	Shape (Configuration) fitting	
SF Index	Principle Component Index		Mode number	Wave number (frequency)	Power (exponent)	Control point/parameter index	
SF Types	Nonlinear System	Linearized System	Not utilized in Soft Robotics	Harmonic (Trigonometric)	$C^{\infty }$ Continuous polynomials	Lagrange polynomials	Hermite, Bezier, PH, BSplines, NURBS
States	Element’s deformation & velocities	Element’s deformation & velocities	Modal participation coefficients	Modal participation coefficients	Polynomial coefficients	Control points’Cartesian states	Fitting method control parameters (e.g., cartesian points and local slopes)
Reported Error	0.3%–3% dynamic	24.6% dynamic	0.7% dynamic (hyper-redundant robot)	NA	1% 2D static loading 9%–19% 3D static loading 1%, no twist dynamic 5.5%–14% 3D dynamic loading	6%–8% static 16%–20% dynamic	3.1–4.1 mm (1%–2%) static (Quintic PH is the best)
Controller	Jacobian inverse	Jacobian inverse	Modal excitation Energy shaping	Feedback linearization	Feedback linearization, Jacobian inverse	-	Jacobian inverse Potential field planning
Material Mech.	Elastic (co-rot. linear), Hyperelastic(neo-Hookean,Mooney-Rivlin)	Elastic (Hooke′s law)	Elastic (Hookes law)	Equivalent elastic elements (e.g. spring/dampers)	Elastic (Hooke′s law)	Elastic (Hooke′s law)	Elastic (Hooke′s law)
Software	SOFA Soft Robotics Toolkit	SOFA Soft Robotics Toolkit	-	-	TMTDyn (shape fitting), SoRoSim (curve fitting)	-	-
Pros	+ High accuracy +3–100× faster than original FEM model	+ Faster than FEM + Can be real-time	+ Theoretical clarity + Identifies system natural dynamics	+ First mode is equivalent to Constant Curvature kinematics + Convergence guarantee	+ Simple state space parametrization + Minimal theoretical & numerical complexity	+ State space similarity to the task space + Easy states’ tracking	+ State space can be similar to the task space + Easy states’ tracking
	+ Can be real-time			+ Real-time (Fast convergence in <5 iterations) + Computationally efficient	+ Convergence guarantee + Real-time + Dynamic implementation + Discontinuity via Spline + Curvilinear frame tangency guarantee vie Bishop’s frame	+ Real-time	+ Real-time + handles discontinuity (mixed curve-straight segments)
Cons	- Needs a prior FEM model - Prior FEM can be computationally expensive - Complex states without physical meanings	- Needs a prior computationally expensive FEM model - Linearization inaccuracy - Complex states without physical meanings	- Suitable for simple systems - Nontrivial nonlinear modal analysis technique (e.g., mode manifold stabilization)	- Nontrivial basis functions - Integration issues for Variable Curvature kin	- Solution sensitive to Runge’s phenomenon - First order solution is parabolic (similar to an Euler Bernoulli EB beam) and not a Constant Curvature - Inaccurate local frame tangency compliance for Frenet-Serret frame	- Overfitting issue (converges only with special point placement) - Extra consideration is needed for curvilinear frame twist - Complex modes - Suitable for simple/static cases	- Converges only with special control point placement - Extra consideration needed for curvilinear frame twist - Complex modes - Complex fitting procedure (e.g., for PH)
Refs	(Goury, Duriez; Chenevier et al., 2018)	(Coevoet et al., 2017; Thieffry et al., 2018a; Thieffry et al., 2018b; Katzschmann et al., 2019; Thieffry et al., 2019; Thieffry et al., 2020)	(Della Santina et al., 1806; Albu-Schaeffer and Della Santina, 2020)	(Chirikjian, 1992; Chirikjian, 1993; Chirikjian and Burdick, 1994; Chirikjian and Burdick, 1995; Chirikjian, 1997)	(Orekhov and Simaan, 2008; Godage et al., 2011a; Godage et al., 2011b; Godage et al., 2016; Sadati et al., 2019a; Grazioso et al., 2019; Della Santina et al., 2020a; Sadati et al., 2020a; Della Santina, 2020; Della Santina and Rus, 2020; Rao et al., 2021; Rao et al., 2022)	Sadati et al. (2018)	(Singh, 2018; Singh et al., 2018; Mbakop et al., 2021)

**TABLE 3:** State Space Coordination in continuum and Soft Robot modeling.

State space	Reported error	Pros	Cons
Absolute coordinates (states): Shape, Configuration, Cartesian position & orientation, Rectilinear frame, Material frame (Sadati et al., 2018; Singh, 2018; Singh et al., 2018; Sadati et al., 2019a; Sadati et al., 2020a) Finite Element Method (Coevoet et al., 2017; Coevoet et al., 2017; Goury and Duriez, 2018; Thieffry et al., 2018a; Thieffry et al., 2018b; Chenevier et al., 2018; Thieffry et al., 2019; Thieffry et al., 2020)	1%–19% static 5.5%–20% dynamic 0.3%–3% dynamic FEM	+ State space is task space (simpler tracking & path planning control) + No spatial integration is required + Simpler derivation of inertial terms for system dynamics + Simpler derivation of external forces & contacts + Simpler derivation of constraints for parallel mechanisms + Numerically more efficient & stable + Suitable for large systems + lower dimensional state space + Geometrically exact solution via FEM	- Higher polynomial order is required hence more system states - Complex formulation for internal forces and elastic energy
Relative coordinates (states): Curvature, Strain, Curvilinear frame, Spatial frame (Chirikjian, 1993; Chirikjian and Burdick, 1994; Chirikjian and Burdick, 1995; Chirikjian, 1997; Orekhov and Simaan, 2008; Grazioso et al., 2019; Della Santina et al., 2020a; Della Santina, 2020; Della Santina and Rus, 2020; Mbakop et al., 2021; Rao et al., 2021; Rao et al., 2022)	0.7% dynamic (for a hyper-redundant robot)	+ Lower polynomial order is required hence less system states + better clarity in employing rigid-body robotic techniques + Matches internal actuation states resulting in a simpler controller design + Elastic energy has linear formulation	- Requires integration & approximation - Complex dynamics - Numerically expensive & less stable for large systems - Not suitable for large systems
Hybrid states (Sadati et al., 2022)	2.1% stable dynamics 7.8% instable dynamics	+ Guaranteed conformation of local frame to backbone tangent + Stable for simulation of instable motions of a continuum backbone + Lower dimensional state space versus polynomial shape kinematics	- Complex Coriolis term derivation - Numerically expensive

**TABLE 4:** Solution strategies for reduced order modeling of soft robots.

State space	Reported error	Pros	Cons
Ritz (Chirikjian, 1992; Chirikjian, 1993; Chirikjian and Burdick, 1994; Chirikjian, 1995; Chirikjian and Burdick, 1995; Chirikjian, 1997; Sadati et al., 2018; Singh, 2018; Singh et al., 2018; Sadati et al., 2019a; Grazioso et al., 2019; Della Santina et al., 2020a; Sadati et al., 2020a; Della Santina, 2020; Della Santina and Rus, 2020)	1%–19% static 16%–20% dynamic	+ Simpler derivation & implementation + Faster convergence	- Less accuracy - No control over convergence rate
Ritz-Galerkin (Tunay, 2013; Coevoet et al., 2017; Goury, Duriez; Thieffry et al., 2018a; Thieffry et al., 2018b; Chenevier et al., 2018; Sadati et al., 2018; Thieffry et al., 2019; Thieffry et al., 2020)	0.3%–3% dynamic (FEM MOR) 6%–8% static (ROM fitting)	+ can be more accurate + Standard practice in FEM	- More sensitive to numerical errors - Possible divergence issues due to numerical instability - Not investigated for Soft Robots

### 3.1 Reduced Order Modeling (ROM) for kinematic formulation

Cosserat rod formulation of continuum robots results in a set of Partial Differential Equations (PDE) that form a Boundary Value Problem (BVP) in continuous domain. Traditionally, the Cosserat rod BVP formulation is turned into a discretized BVP with finite number of states, e.g., via Pseudo Rigid Body or Reduced Order kinematics, to be solved by an optimization-based (e.g., shooting) numerical method. Alternatively, weak-form solutions can be sought for a BVP, a common practice in applied mathematics and FEM analysis. To this end, the separation of variables method is used to decouple the temporal (time-dependent, i.e., states evolution over time) and spatial (space-dependent, i.e., states evolution along the robot backbone arc length) characteristics. As a result, the system of PDEs is replaced by two decoupled Ordinary Differential Equations (ODE), each with respect to either the spatial (arc length) or temporal (time) variables. Hence, a solution can be provided using an ODE solver (either analytical or numerical). The former method results in a kinematic formulation similar to rigid body robots, while the former approach requires fewer modeling states, hence is more computationally efficient. Seeking a solution via forward integration and minimal state space dimension of a Reduced Order Model weak solution are desirable for Soft Robotics controller design (Della Santina et al., 2110). We have categorized the different weak-form solutions to approximate a soft robot kinematics in Table 2.

A basis function-based (modal and fitting) approach can simply the complexity and high-dimensionality of the state space of a soft robot model via:

1. *Modal approaches* where the system kinematics is approximated by a weighted sum of the dominant deformation modes, or
2. *fitting* where the system kinematics is interpolated based on a basis function with control parameters.

As a result, the time-variant weights (modal contributions) in the modal approach or the control parameters of the fitting approach are the new system modeling states, and the mode shapes or fitting basis function are the spatial (i.e., w.r.t. curve length) basis functions. A basis function-based approach separates the variables of the original PDE system and enables seeking an ODE solution. Similar to the application of this method in flexible-arm robotics (Book, 1990), mode shapes (shape or basis functions) can be found based on:

1. mode shape or basis function pre-selection/design in the form of
  a. spectral (e.g., Fourier) (Chirikjian, 1992; Chirikjian and Burdick, 1994) or power (e.g., Taylor, i.e., simple polynomials or splines) (Sadati et al., 2018; Della Santina et al., 2020a; Sadati et al., 2022) series expansions for a modal approach, and
  b. *configuration* fitting via Lagrange, Hermite Bezier, Pythagorean Hodograph (PH), B-Spline, NURBS polynomial) (Singh et al., 2018) for a fitting approach;
2. eigenfunctions based on an eigenvalue problem (via Singular Value Decomposition) after linearizing the system;
3. experimental-based mode shape (via a modal test) or basis shape function selection based on the actual system tests (Rao et al., 2021; Rao et al., 2022).

Although investigated for flexible-arm robots in the 1980s (Book, 1990), cases (2) and (3) are relatively unexplored for soft robots. The case of the Eigenvector solution to a finite element problem is discussed as a part of Model Order Reduction techniques in the following sections (Book, 1990).

Modal approach results in a truncated series solution (with a finite number of terms that are adequate to achieve the desired simulation accuracy) with a finite number of terms (time-variant coefficient and space-variant basis functions). The fitting approach does not necessarily result in a series solution format as it employs a predefined basis function. In both cases, the solution for the system kinematics should satisfy the system initial (i.e., initial stationary state) and boundary (e.g., right angle at the robot base) conditions. As a result, the approximate solution complies with the overall system kinematics (e.g., configuration (Sadati et al., 2020a) or curvature (Della Santina and Rus, 2020) along a continuum arm backbone), and overall system velocity field. The basis function-based kinematics approximation improves the simulation numerical performance and results in a system of acceptable dimensions for controller design. This way, rather than attacking the general modeling problem directly, a low-dimensional approximate model is introduced and studied in detail (Della Santina, 2020).

Reduced Order Modeling in the form of assumed continuous modes is a common approach in computer graphics for reducing the rendering computational cost of large scenes where modeling accuracy is not a priority (Barbic et al., 2009; Badías et al., 2018). In the robotic community, a similar approach has been taken since the 1980s for modeling and control of flexible-arm (Book and Majette, 1983; Tsujisawa and Book, 1989; Book, 1990; Moberg, 2010; De Luca and Book, 2016) and hyper-redundant under-actuated (Chirikjian, 1992; Chirikjian, 1993; Chirikjian and Burdick, 1994; Chirikjian, 1995; Chirikjian and Burdick, 1995; Chirikjian, 1997) robots. All the reported instances of Reduced Order Modeling in this survey have utilized an elastic material mechanics based on Hooke’s law. In the rest of this section, we summarized the main different Reduced Order formulations presented for a soft robot kinematics.

#### 3.1.1 Modal approach

Modal approach is equivalent to interpolating a precomputed basis (the dominant deformations) to model the run-time pose of an object (Xu and Barbič, 2016). In a FEM-based framework, the complex deformation of a continuum object can be reduced to a weighted summation over a finite-dimensional subset of all possible deformations of an object, which is referred to as the dominant deformation subset (Wang et al., 2015) or assumed modes. Such a method achieves good computational performance but at the cost of accuracy, which can be improved by carefully selecting the dominant deformation subset and increasing the number of terms in the summation series. Unlike mode shapes that are stand-alone analytical solutions from the modal analysis of a system, assumed modes (basis or shape functions), despite satisfying the system BCs, may not represent a modal solution for the system and only provide a basis for approximating the system solution, to be verified numerically. For example, although assuming a polynomial solution for the deformation of a continuum manipulator can provide adequate simulation accuracy and computational efficiency, it is not a solution for the modal analysis of the same system. A modal representation of a soft robot kinematics simplifies the infinite-state-space-dimension of the system variable curvature kinematics and transform it to a finite dimensional representation, i.e., a fitting problem with a few weighting coefficients as the system states, and hence results in less theoretical complexity.

The modal approach is a common method for dynamic modeling of flexible-link robots and structures (Book, 1990; Sayahkarajy, 2018). For example, Wu et al. have investigated nonlinear model order reduction based on the system modal derivatives (Wu and Tiso, 2016) limited to simple geometries. Alternatively, Weeger et al. utilized pre-defined shapes in the form of 4D NURBS curves to express the configuration and quaternion orientation of a Cosserat rod centerline in large deformation (Weeger et al., 2017). Liu et al. utilized a polynomial collocation spectral method (in which the series index is the basis function wave numbers, i.e., frequency in the spatial domain) for a Kirchhoff’s rod discretized on a set of Chebyshev or Legendre Gauss-Lobatto control points (Liu et al., 2018). They showed the spectral method precision exponentially increases as the number of the nodes increases.

Modal approaches have been preliminarily investigated for Soft Robots in the form of run-time fitting techniques based on a precomputed basis (i.e., assumed deformation modes) as presented by Wang et al. (Wang et al., 2015) and Xu et al. (Xu and Barbič, 2016). Della Santina et al. (1806) utilized a robot with compliant joints stabilized via sub-manifolds on the system state space (i.e., system nonlinear continuation of linear eigenspaces, called nonlinear normal modes) on which the system would naturally evolve for motion generation. Alternatively, one could bypass the constitutional and conservational laws (system and material mechanics) by matching the basis functions from the experimental observations as the ground truth as presented by Chirikjian et al. for modeling volume preserving 3D deformations of soft simple geometries (Chirikjian, 1995). A similar method has been presented for characterization of worm locomotion kinematics (Digumarti et al., 2019). SOFA (Software Open Framework Architecture), as a computationally efficient simulation tool for evaluating random deformations of soft objects, can serve as an ideal platform in such method to identify an object’s dominant deformation basis.

#### 3.1.2 Polynomial shape (PS) fitting for configuration space characterization

Amongst the early works on Reduced Order Modeling in Soft Robotics, the shape fitting concept was utilized by Godage et al. since 2011 as a singularity free representation of a continuum manipulator kinematics in which the backbone Cartesian configuration is expressed in terms of a power series with basis functions based on a manipulator actuation chambers length (Godage et al., 2011a; Godage et al., 2016). However, their representation could not capture the backbone twist and the choice of a hard-to-interpret basis function resulted in a series solution with many coefficients to identify (e.g., 165 (Godage et al., 2011a) and a complicated system mechanics derivations (Godage et al., 2011a; Godage et al., 2011b; Godage et al., 2016). Sadati et al. showed the advantageous numerical performance and accuracy of using a simpler alternative based on parametrization of the configuration space, i.e., the robot backbone shape (Sadati et al., 2018). A truncated Lagrange polynomial passing through a number of equally spaced control points was used to describe a continuum manipulator shape (configuration or kinematics) with the Cartesian position of the control points as the system modeling states. This method was referred to as Polynomial Shape fitting for soft robot kinematics.

Although it could successfully handle hyper-elastic deformation of the robot cross section, considerations for braided pneumatic actuators, and general loading cases for planar motions, the use of the Frenet-Serret tangent frame formulation invalidates the 3D solutions in the case of a twisted backbone due to external loading. The issue lies with a non-physical twist predicted based on the mathematical definition of a Frenet–Serret curvilinear frame. The same issue presents itself in the work by Singh et al. (Singh, 2018; Singh et al., 2018) and utilized by Wiese et al. (Wiese et al., 2019). Higher modeling and control accuracy could perhaps be achieved by considering this effect, noting that neglecting to do so may result in severe errors in the case of out-of-plane bending. Furthermore, the convergence of a Lagrange polynomial series solution (i.e., solution accuracy increase by the addition of control points) is not guaranteed with a set of equally-spaced points (Sadati et al., 2018). This is due to Runge’s phenomenon (oscillation problem of the fitted curve at the ends of an interval) that can be addressed by using Chebyshev or Legendre Guass-Lobatto techniques (Liu et al., 2018).

To address these issues, a general 3D method is presented in (Sadati et al., 2019a; Sadati et al., 2020a) in which a 7D series solution (i.e., with seven independent series, three for the cartesian coordinates vector and four for the orientation quaternion vector) represents a continuum rod backbone’s 3D Cartesian position and quaternion orientation. This approach is similar to that of (98) but employs simple polynomials instead of NURBS curves. Instead of using Cartesian locations as system states as in (12), the new method uses the polynomial coefficients themselves, thereby improving convergence otherwise affected by Runge’s phenomenon. This also simplifies the derivation complexity caused by the nonlinear form of Lagrange polynomials.

However, in the 7D solution, the conformity of the local frame to the robot backbone tangent vector is governed by the system conservational law (i.e., compliance potential field). These constraints may not perfectly hold for a coarse spatial integration step. The exact formulation of the deformed frame twist (or local curvilinear frame in general) is needed for modeling instability-induced rapid snapping motions that are observed in some continuum robots such as Concentric Tube Robots (Mitros et al., 2021). This is addressed based on a Bishop’s frame formulation for the tangent frame in (55) by introducing a correction twist on an arbitrary tangent frame to the robot backbone. As a result, the system kinematics can be described by a 4D series solution (three for the cartesian coordinates and one for the correction twist) at the cost of slightly more complex derivation of the governing equations. A spline may be used instead of a polynomial when certain degree of continuity is required along the robot overall backbone, e.g., due to the change in the robot structure as in a Concentric Tube Robot. The transition between the spline segments can be continuously implemented based on logistic functions. This representation is used to present the first dynamic model of an everting growing (vine) robot in 3D motion (Berthet-Rayne et al., 2021). The dynamic modeling and controller design of hybrid rigid-soft robots based on the Polynomial Shape kinematic are implemented in an open-source Matlab software package, called “TMTDyn” (Sadati et al., 2020a).

Although being the most common choice, polynomials aren’t the only options to describe the shape of a soft robot. Singh et al. compared Hermite, Bezier, Pythagorean Hodograph (PH), BSplines, NURBS curves for this purpose and showcased, in simulation and experiments, the advantages of a PH curve representation for the controller design of a continuum arm (Singh, 2018; Singh et al., 2018). This representation is later adopted for potential field-based obstacle avoidance of continuum arm on a mobile platform while navigating an unconstructed environment (Mbakop et al., 2021).

#### 3.1.3 Polynomial curvature (PC) fitting for curvilinear space characterization

Amongst the early works, shape approximation techniques were more common to model flexible arm robots (Book, 1990). On the other hand, polynomial approximation of the strain (curvature and torsion) along a continuum backbone (Polynomial Curvature kinematics) was investigated to model planar motion of a hyper-redundant robot and later a DNA strand by Chirikjian et al. (Chirikjian, 1992; Chirikjian, 1993; Chirikjian and Burdick, 1994; Chirikjian and Burdick, 1995; Chirikjian, 1997) who referred to it as “Modal Approach.” It is introduced to constrain a hyper redundant arm curvature to a representation with a few DOFs based on trigonometric (sine and cosine) functions as the assumed mode shapes for the curvature values, and time-variant coefficients as the modal participation factors.

In soft robotics research, Polynomial Curvature kinematics and Lagrange dynamics of a planar Kirchhoff rod (i.e., infinite shear) were studied by Della Santina et al. (who, for the first time, referred to it as Polynomial Curvature kinematics) highlighting their advantages and challenges in controller design (Della Santina et al., 2110; Della Santina and Rus, 2020; Della Santina et al., 2020a; Della Santina, 2020). A 1D series solution was used for the curvature of a planar beam backbone to overcome the robot vibration and reduced performance associated with a Constant Curvature-based controller. A similar method with Cosserat rod mechanics was studied for quasi-static 3D modeling of continuum rod in (27) focusing on the compatibility of the curvilinear constraints such as those seen in a Concentric Tube Robots (Sears and Dupont, 2006; Webster et al., 2006; Mitros et al., 2021). More recently, 3D implementation of the Polynomial Curvature kinematics is showcased and verified in standard simulation scenarios by Boyer et al. (Boyer et al., 2020), who referred to their presented kinematics as “Piecewise Variable Curvature”. There, the problem of deriving the complex governing equations for the system dynamics is addressed based on a recursive computational scheme which they referred to as “Inverse Dynamic Model.” Later, this method is showcased for modeling closed-chain (parallel) soft robots (Armanini et al., 2021) and made available in an open-source Matlab software package, called “SoRoSim” (Teejo Mathew et al., 2021).

Alternatively, the right basis function candidate can be identified based on the fitting performance of different shape functions to capture the shape of a real soft robot. As an instant of Polynomial Curvature method, Rao et al. have demonstrated that an Euler curve (a curve whose curvature varies linearly along the arc length) is a viable candidate to capture the planar deformation of a continuum appendage under external tip load (Rao et al., 2021; Rao et al., 2022). Similarly, modal test results on the actual soft robots can be used to construct the basis functions for the robot’s reduced order kinematics, as practiced for flexible arm robotics in 1980s (Book, 1990).

The curvilinear space, i.e., strains (shear, elongation, curvature, and torsion) requires one fewer state compared to the rectilinear space (i.e., absolute Cartesian position and orientation) to describe continuum backbone kinematics, since the curvilinear space is derived through spatial differentiation from the rectilinear parameters. However, deriving the rectilinear parameters is still required for calculating the local effect of external loading and the system inertial dynamics. This requires spatial integration of a set of non-integrable trigonometric functions. These integrations are handled via analytical approximation (e.g., Taylor series expansion (Della Santina, 2020; Della Santina and Rus, 2020)) of the integrands (Della Santina et al., 2020a; Della Santina, 2020; Della Santina and Rus, 2020) or numerical integration methods (Orekhov and Simaan, 2008). The former method is complex and inaccurate for large deformations (outside the Taylor series convergence radius) but necessary for integration in Lagrangian dynamics. The latter method is only compatible with optimization-based solutions (e.g., shooting method). More accurate solutions for these integrals are proposed based on the formulation of the closed-form integrals with 0th-order Bessel functions for the 2D case (Chirikjian, 1992) or, more generally, the use of integrable basis functions such as helical functions (Grazioso et al., 2019). The Polynomial Shap approach overcomes these limitations (i.e., integration complexity, inaccuracy, and lack of generality) by relying on a differentiation step to obtain the curvilinear space parameters (required to calculate the strain actions) instead of the integration step required by the polynomial Curvature method to obtain the rectilinear space parameters.

However, unlike the Polynomial Shape method, the definition of curvilinear frame is not problematic in the case of Polynomial Curvature method due to the direct parametrization of curvature and torsion. The only exception is the method presented by Grazioso et al. (Grazioso et al., 2019) in which the deformation map derivation relies on a curve in the Frenet-Serret frame that is constructed by analytical integration of helical basis functions for the curvilinear (curvature) space. There. similar to the works by Singh et al. (Singh, 2018; Singh et al., 2018), the problem with an deformed local frame twist isn’t addressed.

Most of the recent Polynomial Curvature related studies are limited to theoretical investigations (Orekhov and Simaan, 2008; Grazioso et al., 2019; Della Santina et al., 2020a; Della Santina, 2020; Della Santina and Rus, 2020). Future experimental studies on soft robot hardware and a comparison with Polynomial Shape methods can help to achieve a fair comparison and conclusion on the most appropriate method for a given Soft Robotics application.

### 3.2 Model Order Reduction (MOR) for finite element models

In this paper, we used the Reduced Order Modeling term for the techniques that provide an approximate solution (usually in a basis function-based from) for the system kinematics. The system kinematics and material mechanics are then fed into the system mechanics to derive the system’s equation of motions. Alternatively, we employed the term Model Order Reduction (MOR) to refer to the techniques that reduce the state space dimension of an already developed model (usually based on FEM) for a soft system. Although Reduced Order Modeling and Model Order Reduction are employed interchangeably in some literature, the reason for the use of different terms in this paper is that Model Order Reduction, unlike Reduced Order Models for a robot kinematics, is not a fundamental block (i.e., the system kinematics, system mechanics, and material mechanics) of a continuum robot model. Model Order Reduction is also referred to as System Compression.

The Model Order Reduction utilizes Principal Orthogonal Decomposition or Principal Component Analysis techniques which are widely used in computational mechanics. There, Singular Value Decomposition is performed on the state variables of several sample deformations of a soft robot (minimum 2 within each actuation range). The results are truncated up to a tolerance to define the system reduced basis and to neglect insignificant system states. The insignificant states are hard to reach (require large excitation energy) or hard to observe (produce small observable energy) (Antoulas, 2005). Such basis need not necessarily have any physical meaning. Measures are considered to achieve desired tolerance while preserving the system properties such as stability, passivity, and contractility. The compressed (reduced) system can be used for nonlinear controller and observer design with stability guarantee (Chenevier et al., 2018).

Principle Orthogonal Decomposition, although being the only technique suitable for a nonlinear system (Thieffry et al., 2018a), is computationally expensive and therefore not real-time. To achieve real-time performance, Thriftey et al. (Thieffry et al., 2018a; Thieffry et al., 2018b; Thieffry et al., 2019; Thieffry et al., 2020) performed the same analysis on a linearized FEM model around an equilibrium point, and utilized their reduced system to formulate a stable observer and controller. As a result, their reduced model was 3–100 times faster in simulations than the original FEM model. Alternatively, Goury et al. (Goury, Duriez) computed a reduced integration domain (and weights associated with it) by computing the reduced basis on the sample deformation space and simultaneously storing the elements contributions based on an assumed tolerance. The Galerkin method was then used to solve the resulted system. Elastic material mechanics based on co-rotational linear model (Chenevier et al., 2018) and hype-elastic material mechanics based on neo-Hookean (Goury, Duriez; Chenevier et al., 2018) and Mooney-Rivlin (Goury, Duriez) models have been utilized in different Model Order Reduction implementations in the literature.

Model Order Reduction method is implemented in the “Soft Robotics Toolkit,” an open-source simulation package based on SOFA-framework. This implementation benefits from the real-time performance, open-source (hence expandable), accurate, and configurable numerical methods of SOFA and is experimentally verified for nonlinear controller design of soft robots of general shape (Goury, Duriez; Thieffry et al., 2018a; Bieze et al., 2018; Thieffry et al., 2018b; Thieffry et al., 2019; Thieffry et al., 2020; Katzschmann et al., 2019). However, the underlying FEM model results in high computational cost, lack of theoretical clarity (i.e., non-interpretability of the modes and complex nonlinear formulation), and complexity in implementing new assumptions (e.g., constraint) and elements (e.g., braided pneumatic actuators). Furthermore, the current implementation of SOFA-framework cannot handle Coriolis terms in dynamics of rigid bodies and hence poses difficulty in modeling hybrid rigid-continuum body structures. The plugin enables implementing a variety of material laws that are already available in SOFA.

### 3.3 State space coordination and dimensionality

A continuum system can be modeled based on states defined in relative (i.e., the continuum manipulator local tangent frame) and absolute (i.e., ground fixed reference/inertial frame) coordinates, which are called “relative states” and “absolute states,” respectively, in the rest of this paper. Relative states are usually referred to local strain or free parameters of a fitting method that describes a single segment w.r.t. the previous segment (local frame) in a series chain (e.g., in the case of Constant Curvature case). An absolute state refers to the robot shape itself (e.g., node position and quaternion orientation), participation coefficients of the robot shape modal representation, or free parameters of an fitting method that describes a robot segment w.r.t. the global frame (e.g., Hermite, Bezier, PH, or NURBS fitting of a robot backbone shape). Recently, a hybrid-state model is presented that combines the absolute (e.g., position) and relative (e.g., local correction twist angle to represent a Bishop’s frame) states (e.g., in the case of Concentric Tube Robots) (Sadati et al., 2022).

Except for FEM research, relative state representations are dominant for modeling continuum robots due to simplicity in dealing with system elastic energy, internal forces, and actuation inputs. Modal representations with relative states require lower order basis functions too, e.g., a polynomial curvatures representation relies on 2^nd^-order polynomials while polynomial shape representations require a 3^rd^-order polynomial to capture the backbone basic motion and satisfy the boundary conditions (e.g., backbone right angle at the robot base). However, implementation of external force fields, contacts, and constraints are challenging. Furthermore, the system dynamic derivation involves an integration stage to account for the inertial effects that is usually dealt with based on approximate numerical solutions that may reduce the overall accuracy and increase the numerical cost of the system simulation. The complexity of the equations of motion increases as the number of elements, states, and segments in a system increases, making them unsuitable for large-scale systems. The similarity of such representations with the models for a rigid-link serial mechanism makes it easier to utilize well-developed techniques from rigid-body robotics. As a result, relative states are favorable for control system design in continuum robotics research.

On the other hand, absolute states result in an easier derivation of inertial terms, external forces, contact, and constraint (e.g., for parallel mechanisms). The system dynamic derivation involves a differentiation stage that can be accurately handled analytically, and the equation of motion derivation complexity does not significantly increase as the number of elements in the system increases, making it suitable for large-scale systems. As a result, absolute states are favored for FEM modeling in the Soft Robotics community. Furthermore, considering the robot shape in task space (configuration in global frame), as the system absolute states, makes such methods more suitable for trajectory tracking, shape and/or tip motion control.

Absolute representation of position and orientation does not kinematically constrain the local frame to the backbone curve tangent and this should be enforced by the governing equations, i.e., due to balance of shear and bending virtual energies. A hybrid state space method is recently proposed that benefits from absolute states for the robot position while defines a relative correction twist angle to define a local Bishop’s frame based on an arbitrary tangent frame to the resultant curve. As a result, for the first time, a seamless framework is derived that can handle instability instances in the dynamics of a continuum system, in this case a Concentric Tube Robot (Sadati et al., 2022). Such a hybrid presentation results in a more complex derivation of the Coriolis terms due to the need for derivation of the backbone curve tangent frame. However, it benefits from a smaller state space since the orientation is defined based on a single correction twist angle instead of three states for Euler angles or for states for a quaternion representation.

In the case of geometrically exact modeling, variable Curvature kinematics result in an infinite-dimensional states space based on strains (relative states) along the robot backbone. Alternatively, FEM and Absolute Nodal Coordinate Formulations, which result in a large-dimensional state space based on only position (FEA (Coevoet et al., 2017) or both position and orientation (Absolute Nodal Coordinate Formulations (Shabana, 2018; Huang et al., 2021; Shabana and Eldeeb, 2021) of a finite number of nodes along the robot backbone, are instances of cases with absolute states. On the reduced order modeling front, Constant Curvature assumptions, modal, and fitting-based representation of a continuum backbone strain domain deal with relative states. On the other hand, modal, and fitting-base approximation of the robot shape present a reduced order representation of a system with absolute states. Recent formulation of a Concentric Tube Robot kinematics based on polynomial shape approximation in reference coordinates for the backbone position and Bishop’s frame representation for orientation in relative coordinates is an example of hybrid state formulation. The main features, advantages, and shortcomings of different state space coordination employed in continuum and Soft Robotics research are summarized in Table 3.

### 3.4 Solution strategies

The resulted approximate solution for the system kinematic or mechanics based on reduced order modeling or model order reduction is called a weak solution in continuum mechanics and applied mathematics. Such solution is then substituted in the system governing equations. Afterwards, the Ritz or Ritz-Galerkin method can be used to solve the resulting system (Hesthaven et al., 2007).

Substituting an approximate (e.g., basis function-based) solution for the system deformation map. i.e., kinematics, in the system governing equations is called the Ritz approach to solve a system of complex PDEs. Although it is not referenced in most Soft Robotics research works, most of the proposed models for soft robots based on a basis function based approximation utilize the Ritz method. Later, the spatial domain of the resulted decoupled system can be numerically integrated while the time-varying coefficients are optimized to minimize the governing equation residual error.

Alternatively, the coefficients can be considered as the system’s EOM states for which the time series can be found from the numerical integration in time (Sadati et al., 2018). The Galerkin method of weighted residuals, known as Ritz-Galerkin method, may provide a better approximation where the weighted residual of the system is minimized instead of the residual function itself (Tunay, 2013). In simple words, both sides of the governing equations resulted from the Ritz method is multiplied by a vector, called weighting vector, which usually consists of the shape (basis) functions. Tunay et al. (Tunay, 2013), Sadati et al. (Sadati et al., 2018), and Goury et al. (Goury, Duriez) have investigated Ritz and Ritz-Galerkin solutions for continuum robots in the soft robotics community. Table 4 summarizes the main features of these solution strategies.

### 3.5 Controller design implications

Standard control architectures (e.g., Jacobian-based inverse dynamics (Coevoet et al., 2017; Goury, Duriez; Thieffry et al., 2018a; Thieffry et al., 2018b; Katzschmann et al., 2019; Thieffry et al., 2019; Thieffry et al., 2020), feedback linearization (Della Santina et al., 2020a; Sadati et al., 2020a; Della Santina and Rus, 2020), potential field planning (Mbakop et al., 2021) for finite-state space systems, such as rigid body robots, have been integrated with Reduced Order models of soft manipulators (see Table 2). From the formulation perspective, the use of absolute states with simpler mapping between the control system states and the robot workspace (e.g., shape position and orientation) may simplify the controller design for tracking and planning scenarios in the robot task space. On the other hand, the use of relative states, with a simpler mapping between the system states and the actuation inputs, may simplify the configuration space controller design. This is useful for tasks involving vibration attenuation or under-actuated system control design. A detailed discussion on model-based control of soft robots is presented in (9). A detailed comparative study of different control strategies for Reduced Order Model of soft manipulators is needed to better highlight the performance of different combinations of the Reduced Order modeling and control technique.

### 3.6 Comparative studies and software packages

To reach a fair comparison between the different methods in the literature, it is important to minimize the effect of experimental hardware and procedures on the results between different studies. To this end, comparative studies of different methods based on experimental results with a unique setup can be helpful. We have conducted a series of such comparative studies based on experiments with a soft appendage with pneumatic braided actuators, known as STIFF-FLOP (STIFFness controllable Flexible and Learn-able Manipulator for surgical OPerations) in (Sadati et al., 2017a; Sadati et al., 2018; Sadati et al., 2019a; Sadati et al., 2020a). A more recent study by Rao et al. (2020) compared the accuracy of different modeling techniques (Constant Curvature, Pseudo Rigid Body, Piecewise Constant Curvature, and Variable Curvature) for a tendon-driven continuum robot. To help with choosing the right elements for modeling a soft manipulator, Table 5 provides a summary of findings based on these comparative studies.

**TABLE 5:** Summary of the findings from comparative studies of different modeling methods as presented in (Sadati et al., 2017a; Sadati et al., 2018; Sadati et al., 2019a; Sadati et al., 2020a; Rao et al., 2020).

Kinematics	Mechanics (conserv. Law)	Advantages	Disadvantages
*Pseudo Rigid Body*	*Lagrangian Dynamics*	+ Good accuracy and numerical performance + Suitable for dynamic analysis and traditional control design	- Numerically expensive for complex systems - Sensitive to incorporating nonlinear material laws - Suitable for simple systems with a small number of states (less than five discretized segments) - Suitable for cases without external loading
*Constant Curvature*	*Any model*	+ Suitable for incorporating structural complexity + Suitable for formulating a design parameter study	- Not accurate for hyper-elastic structures - Not accurate in the presence of body and external loads
*Piecewise Constant Curvature*	*Lagrangian Dynamics*	+ Suitable for dynamic analysis and traditional control design + Favorable for stiff continuum systems	- Numerically expensive for complex systems - Sensitive to incorporating nonlinear material laws - Favorable only for a fine-tuned number of segments ( QUOTE ) but loses accuracy for smaller and numerical efficiency for a larger number of segments
*Variable Curvature*	*Cosserat Rod*	+ Advantageous for accuracy in general loading cases with a large number of nodes	- Slow numerically expensive simulations for general boundary condition cases
*FEM & Absolute Nodal Coordinate Formulation*	*Lagrangian Dynamics*	+ Most computationally efficient for complex systems + Suitable for incorporating complex material mechanics models	- A large number of modeling states resulting in computationally expensive simulations - Complex controller design due to a large number of states
*Data-driven* (robot shape as a function of the actuator inputs)	*NA*	+ Suitable for real-time performance + High accuracy for the learned dataset	- No stability guarantees - Lack of accuracy in the case of extrapolation (i.e., cases out of the learned dataset)
*Reduced Order* (*modal & shape fitting* via 2nd-order *polynomial & Euler curves*)	*Principle of Virtual Work*	+ Combines most of the above advantages, i.e., accuracy, simple controller design, near real-time performance, incorporation of structural complexity, and general loading cases + Numerically robust performance	- Complex implementation for general boundary conditions

There has been an increasing interest in the application and contribution of reduced order basis function-based methods handling singularity issues (Godage et al., 2011a; Godage et al., 2011b; Godage et al., 2015a; Godage et al., 2016), general loading cases (Sadati et al., 2018; Sadati et al., 2019a; Sadati et al., 2020a; Rao et al., 2021; Rao et al., 2022; Sadati et al., 2022), kinematic constraints (Orekhov and Simaan, 2008), force observation (Aloi and Rucker, 2019; Aloi et al., 2022), controller design (Singh et al., 2018; Della Santina et al., 2020a; Della Santina, 2020; Della Santina and Rus, 2020), and path planning (Mbakop et al., 2021) emphasizing the ever-increasing importance of Reduced Order Modeling methods in the Soft Robotics community.

Tailored software packages are developed mostly for FEM-based (Naughton et al., 2009; Coevoet et al., 2017; Gazzola et al., 2018; Sadati et al., 2019b; Grazioso et al., 2019; Hu et al., 2019; Mathew, Hmida, Renda; Huang et al., 2020; Li et al., 2020; Munawar et al., 2020; Bern and Rus, 2021; Graule et al., 2021) implementations targeting fast simulation of complex scenarios for realistic graphical representations and learning-based research. The exceptions are TMTDyn (based on the TMT method) (Sadati et al., 2019b; Sadati et al., 2020a) and SoRoSim (based on a Recursive Computational Scheme) (Mathew, Hmida, Renda), which are Matlab packages for the theoretical derivation of a continuum robot Lagrangian dynamics suited for theoretical dynamical system analysis and nonlinear controller design. Although, similar functionalities have been or can be achieved by further developments around FEM-based packages such as SOFA (Coevoet et al., 2017), PyElastica (Naughton et al., 2009), and DiffPD (Ma et al., 2021). Ease of use, integration with existing tools, and suitability to research objectives alongside excellent accuracy, robustness, and real-time computational performance are key to ensuring wide acceptability.

## 4 Conclusion and future directions

Reduced Order Modeling and Model Order Reduction techniques have been extensively investigated to obtain models of acceptable dimension for Soft Robotics control. In this paper, we reviewed and compared the techniques identified in the literature, summarizing our findings in Tables 2, Table 3, Table 4, Table 5. Although, Reduced Order Modeling and Model Order Reduction techniques are promising paths towards Soft Robotics control, they require further theoretical development, experimental validation, and comparative study.

The quest for finding a better shape fitting technique for soft manipulators is still ongoing with recent studies on Euler curves and Magnus expansion technique. Modeling complex soft manipulators with parallel structure, braided, growing, and concentric elements are the most recent trends in relevant modeling research. On the control research front, application of shape fitting techniques for shape control, path planning, and obstacle avoidance have been among the most recent studies.

Amongst the Reduced Order Modeling techniques, the Polynomial Shape method has been primarily investigated for modeling complex continuum systems, such as Concentric Tube and Eversion Growing Robots. On the other hand, the Polynomial Curvature method has been mostly studied for the control problem of simple continuum manipulators. Both the Polynomial Shape and Polynomial Curvature methods for soft manipulators are now supported by open-source software, dynamical implementation of general deformation and loading cases, experimental studies, and controller designs, but require further theoretical developments. Such developments need to focus on integrating nonlinear material mechanics laws, simulation stability analysis for modeling hyper-elastic material models, and further experimental investigations. On the other hand, Model Order Reduction implementations for soft robots, which relies on a preliminary FEM model, will benefit from more collaborative work, dissemination, and support for implementation by experimentalists.

Immediate next research steps are simulation numerical stability analysis for high bandwidth and unstable rapid motions of soft manipulators, high-bandwidth motion and vibration attenuation control, force and stiffness regulation, external force and impact observation, multi-arm coordination, robot planning for self-collision and obstacle avoidance, and path following based on techniques such as follow-the-leader. The Polynomial Shape and Curvature methods are yet to be explored for parallel structure and path planning problems respectively. Finally, the potential of run-time fitting techniques based on modal test results on the actual system, or a precomputed basis (normal nonlinear modes) are untapped.

In the long run, it is interesting to investigate the Reduced Order Modeling techniques for the emerging research directions in the Soft Robotics such as multi-physics simulations (e.g., soft swimming or flying robots), machine learning and data-driven techniques, and task automation. Finally, developing software toolkits and experimental comparative studies are essential to broaden the reach and impact of the Reduced order Modeling techniques and to help identify each method’s potential from an application-oriented and control perspective.
